# Predictive validity of a new integrated selection process for medical school admission

**DOI:** 10.1186/1472-6920-14-86

**Published:** 2014-04-23

**Authors:** Paul L Simpson, Helen A Scicluna, Philip D Jones, Andrew MD Cole, Anthony J O’Sullivan, Peter G Harris, Gary Velan, H Patrick McNeil

**Affiliations:** 1 Faculty of Medicine, University of New South Wales, Sydney, Australia; 2 School of Public Health & Community Medicine, Faculty of Medicine, University of New South Wales, Sydney, Australia; 3 School of Medical Sciences, Faculty of Medicine, University of New South Wales, Sydney, Australia; 4 South Western Sydney Clinical School, Faculty of Medicine, University of New South Wales, Sydney, Australia; 5 Department of Rheumatology, Liverpool Hospital, Locked Bag 7103, Liverpool BC NSW 1871, Australia

**Keywords:** Student selection, Predictive validity, Predicting, Performance, Demographics

## Abstract

**Background:**

This paper is an evaluation of an integrated selection process utilising previous academic achievement [Universities Admission Index (UAI)], a skills test [Undergraduate Medicine and Health Sciences Admission Test (UMAT)], and a structured interview, introduced (in its entirety) in 2004 as part of curriculum reform of the undergraduate Medicine Program at the University of New South Wales (UNSW), Australia. Demographic measures of gender, country of birth, educational background and rurality are considered.

**Method:**

Admission scores and program outcomes of 318 students enrolled in 2004 and 2005 were studied. Regression analyses were undertaken to determine whether selection scores predicted overall, knowledge-based and clinical-based learning outcomes after controlling for demographics.

**Results:**

UAI attained the highest values in predicting overall and knowledge-based outcomes. The communication dimension of the interview achieved similar predictive values as UAI for clinical-based outcomes, although predictive values were relatively low. The UMAT did not predict any performance outcome. Female gender, European/European-derived country of birth and non-rurality were significant predictors independent of UAI scores.

**Conclusion:**

Results indicate promising validity for an integrated selection process introduced for the Medicine Program at UNSW, with UAI and interview predictive of learning outcomes. Although not predictive, UMAT may have other useful roles in an integrated selection process. Further longitudinal research is proposed to monitor and improve the validity of the integrated student selection process.

## Background

Coinciding with a significant rise in demand for medical education has been increasing scholarly and public interest about how to best select medical students. The traditional use of prior academic achievement for medical student selection has been challenged in recent decades on the basis that it unfairly advantaged students from higher socio-economic backgrounds and excluded many students who would likely become exceptional medical practitioners [1,2]. Since the late 1990s the use of three selection tools has become common practice among Australian medical schools to address such concerns [1,3]. These tools assess prior academic achievement, cognitive skills and personal characteristics as measured through secondary school academic ranking, Undergraduate Medicine and Health Sciences Admissions Test (UMAT) and a structured interview, respectively.

A critical issue noted in the literature is establishing the validity of the UMAT and interview [1,4-6]. Prior academic achievement is the strongest predictor of academic performance at university for a range of disciplines including medicine [7-10]. Studies have shown mixed results regarding the ability of prior academic achievement to predict performance in the later years of medicine [5,10-12].

The UMAT is a test to measure cognitive skills and an understanding of people. UMAT annual reports indicate good internal reliability and validity (Australian Council for Educational Research - ACER, 2011), and while good construct validity has been reported for the UMAT, sections measuring cognitive abilities [13] and its predictive validity have been disappointing [5,14]. However, some assert that UMAT’s value lies more in predicting medical internship and practice performance, thus more longitudinal studies are called for [14].

Studies looking at interview validity have shown mixed results: one study reported low predictive validity yet interview scores predicted Year 4 clinical-based outcomes better than prior academic achievement [14], whilst another found the communication skills dimension predicted performance in the later years of the program specifically clinical skills [5]. Recent studies looking at interview validity have largely focused on the Multiple Mini-Interview (MMI), which has been found to predict clinical skills in later years of the program [15]. Due to resource costs and mixed validity results, the interview remains a controversial method [6]. While some Schools in Australia, like the University of Queensland have abandoned its use [16], others claim such a move is premature [1,17]. Some scholars argue that non-cognitive assessments such as the interview add value to academic criteria thus justifying its use as part of the selection process [3,5,18], while others have reported its value in introducing changes relating to student demographics in terms of gender balance and ethnicity [19].

A lot of the focus on UMAT and the selection interview is, understandably, framed by efforts to achieve high predictive validity. However, efforts to improve predictions of student performance may meet with various degrees of success as a function of the original purpose of the predictive variable of interest [11].

The UMAT and interview were originally included in UNSW’s selection process to serve as tools to distinguish between applicants who meet the criteria of a high UAI, to differentiate students with variable motivations to study medicine (e.g. expose those motivations that stem from social pressures, family expectations), to include assessment of non-academic capabilities, and to enhance and preserve student diversity. Thus, an important original reason for employment of the UMAT and interview at UNSW was based on the rationality of differentiation, rather than predicting specific program outcomes, whilst recognising that validity would be examined at a later stage when data and resources permitted. As a consequence, this original function is likely to impact on predictive relationship values. Small sample sizes and range restriction of predictor and criterion variables may also affect the ability to achieve high validity coefficients [20].

Student demographics should also be considered to determine their role in predicting selection tool performance and Medical Program outcomes [21]. Accounting for the effects of demographics on performance outcomes has largely been confined to measurements of ethnicity [11,22-24]. This study examined country of birth, gender and socio-economic measures relating to educational background and rurality.

At UNSW, an integrated multi-dimensional selection system has been adopted in which performance of applicants in each of the three components of UAI, UMAT and interview, are equally weighted in the calculation of a final ranking (see Table 1). During the year prior to entry, applicants submit an application form and structured curriculum vitae, which asks applicants to respond preliminarily to material that would be explored in greater detail at interview, and which requires the applicant’s school to provide a predicted UAI. Interviews are offered based on the applicants’ known UMAT and predicted UAI outcomes. A second round of interviews is held for applicants whose actual UAI outcomes were much better than predicted by their school. Once all interviews are completed, scores for all three variables (actual UAI, UMAT and interview) are standardised and combined to determine a ranked order of offer for places in the UNSW program.

**Table 1:** Predictor variables examined in the study

**Entry scores**	**Demographic variables**
Universities Admission Index (UAI) determined from the aggregate of results in the state Higher School Certificate examinations undertaken by students in the final year of secondary school.	Country of birth
	- European/European-derived
	- East Asian
	- Other (South Asian, Middle Eastern & African)
Undergraduate Medicine and Health Sciences Admission Test (UMAT) scores derived from 3 dimension scores:	Educational background (Secondary school attended)
	- Group 1 includes standard public and private Catholic (systemic) schools
- Logical reasoning and problem solving	
- Understanding people	- Group 2 includes selective public, private Catholic (independent), private non-Catholic religious, and private non-religious schools
- Non-verbal reasoning	
Structured Interview scores derived from six dimension scores:	Rurality (student from geographically remote residential area)
- Communication skills	- Non-rural student
- Motivation	- Rural student
- Empathy of others	
- Self-awareness	
- Responding to diversity	
- Ability to cope with uncertainty	
Final applicant ranking based on a combination of scores from the three components, weighted approximately 1:1:1 for UAI, Interview score and UMAT	Gender
	- Female
	- Male

The interview instrument developed at UNSW is designed around a biographical assessment of the applicant’s life context and experiences which had led to a desire to pursue a medical career, and the motivation for this. Interviewers score an applicant in a range of dimensions, including for example empathy towards others, communication skills, and coping with uncertainty. An identical process is utilised to select students who meet criteria for entry as ‘rural entry’, although these applicants compete for dedicated places reserved for applicants with a rural origin.

This study aimed to evaluate the integrated selection process for medicine introduced at UNSW in 2003 by determining the predictive validity of prior academic performance UAI, UMAT and interview, controlling for student gender, country of birth, educational background and rurality.

## Methods

### Participants

The first two student cohorts assessed using the new selection process and who were successful in entering the new outcomes-based UNSW Medicine Program in 2004 and 2005 [25], were included in the study. Student program outcomes were tracked over six years of study. International and Indigenous students admitted via a different entry pathway were excluded from analyses. Accordingly, data on 318 students (149 from the 2004 cohort and 169 from the 2005 cohort) were analysed. Where students repeated an assessment, their first outcome was used for analysis.

Demographic, UAI, and educational outcome data were also available from 304 students who studied the previous discipline-based medicine program in 2002 and 2003. The 2002 cohort (145 students) were selected through a ranking based on UAI scores alone, whereas the 2003 cohort (159 students) were selected using the new selection process. Analyses of these two cohorts provide some insight into effects of different selection processes for students undertaking the same discipline-based program.

### Predictor variables

Predictor variables included admission scores and demographic variables. The six interview and three UMAT dimension scores were included in the regression model. Dimensions were used because total scores are constituted by different (though probably overlapping) constructs. Interview dimensions include communication skills, motivation, empathy of others, self-awareness, responding to diversity, and ability to cope with uncertainty. UMAT dimensions are verbal reasoning, understanding of people, and non-verbal reasoning.

Table 1 outlines the demographic variables and their categorisations considered for analysis. Demographic variables include: gender; country of birth (COB); educational background; and rurality. COB was firstly categorised into five groups informed by the Australian Standard Classification of Cultural and Ethnic Groups [26]. Due to small numbers of students in some of these groups, groups were collapsed into three broad groups: (i) European (including European colonial-derived countries of Australia, New Zealand and USA); (ii) East Asian; and (iii) Other. The term “European” was used rather than “Caucasian”. This is because Caucasian “white” people are a heterogeneous grouping who, and/or whose parents, may come from such diverse areas as Europe, North Africa, the Middle East and East Asia. Given this, the authors consider that the term “Caucasian” as a classification is better replaced with identifications based on geographic origin and migration history [27].

Educational background derives from the following Australian secondary schools students attended: selective or standard public school (a selective school is a school that admits students on the basis of an academic selection criteria, whereas a standard school has no selection criteria except residential proximity of the student to the school); private systemic or independent Catholic school (systemic referring to a number of schools belonging to a system, whereas independent schools are not part of a system); private Protestant school; private non-Christian religious school and; private non-religious school. These schools were categorised into two ordinal groups reflecting levels of educational advantage as perceived by the wider community [28]. Group 1 consisted of standard public and private Catholic (systemic) schools. Group 2 comprised private Protestant, private Catholic (independent), private non-Christian, private non-religious schools, and public selective schools.

Rurality considers the geographical remoteness of the student’s primary and secondary schools and residential address. Accordingly, students were categorised as either ‘rural students’ or ‘non-rural students’.

### Performance outcomes

Performance outcomes for the 2004 and 2005 cohorts included the Weighted Average Mark (WAM) for the first two years of study (phase 1), third and fourth years of study (phase 2), fifth and sixth years (phase 3) and for the entire Medicine Program (program final). WAM is calculated from all units completed by a student and takes into account its relative weight in the course. WAMs for phase 2 and phase 3 were included in correlation analysis only. Other performance outcomes include ‘clinical skills’ and ‘knowledge-based’ outcomes (for phase 1 and 3). Clinical skills reflect clinical and generic communication skills and physical examination/procedural skills as assessed through an observed structured clinical examination. Knowledge-based outcomes derive from written examinations. Outcome scores were percentile-based rather than grade-based. For the 2002 and 2003 cohorts, performance outcomes examined were average marks at end of years 2, 4 and 6.

### Statistical analysis

To address range restriction and different score variances across 2004–5 cohorts, standardised scores were calculated for UAI, interview and UMAT total scores using standard competition ranking and *Z-*score conversion*.* In standard competition ranking scores of equal value receive the same ranking number, and then a gap is left in the ranking numbers (e.g. 1, 2, 2, and 4). Data were analysed using SPSS (version 22). Demographic and admission data were compared between 2004 and 2005 cohorts using one-way ANOVA and chi-squared analysis.

Pearson correlation coefficients were calculated for admission (total) scores by performance outcomes, as well as for UAI and selected yearly average scores across 2002 and 2003 cohorts who entered the old Medicine Program. Hierarchical multiple regression analyses were performed with interview dimensions scores, UMAT dimension scores, UAI and demographics as predictors. WAM (phase 1 and program final), clinical skills (phase 1 and 3) and knowledge examinations (phase 1 and 3) scores were used as six separate criterion. Data assumptions relating to normality, linearity, homoscedasticity and tolerance were satisfactorily met before running regression analyses. Pearson correlation coefficients were also calculated for UAI by UMAT and interview total and dimension-item subtotal scores in order to examine divergent validity.

Ethics approval from the UNSW Human Research Ethics Committee (reference No. 2011-7-27) was obtained.

## Results

### Student characteristics

For the 2004 and 2005 cohorts, the mean age was 18.5 years and ranged from 16 to 31 years; 54% were female, 46% male. Most were born in countries that were European/European-derived (64%) followed by East Asian (22.6%) and other countries (i.e. South Asian, Middle Eastern or African (13.4%). Forty three per cent attended a public selective high school, 31% private non-Catholic, 13% standard public, 7% private independent Catholic, 6% private systemic Catholic and 1% went to private non-Christian or non-religious schools. In terms of school categories: 81% attended Group 2 schools and 19% went to Group 1 schools. Over three quarters (78%) were non-rural students. No significant differences were found between 2004 and 2005 cohorts for age, gender, COB and rurality numbers. No significant differences in UAI, UMAT and interview total scores were found between 2004 and 2005 cohorts.

Comparison of the first cohort admitted using the new integrated selection process (2003) with those previously admitted using the UAI alone (2002) showed no statistically different changes in socio-demographic characteristics. The mean ages were 18.24 and 17.97 years for the 2002 and 2003 cohorts respectively. Females comprised 56% of accepted students in 2003, compared to 57.3% for 2002 entry. In 2003, 59% were of European descent, 34.5% were of East Asian descent, and 6.5% were born in other countries compared with 53%, 43.2% and 4.0% respectively in 2000–2002. Whereas all students admitted in 2002 achieved a UAI ≥ 99.75, only 42.6% of students admitted in 2003 were above this cut off. The median UAI of the 2003 admitted cohort was 99.65. Thus, the new integrated selection system opened up more than half of the previous places offered on the basis of UAI alone to applicants with a broader range of capabilities, attributes, and abilities, without major changes to socio-demographic characteristics. Furthermore, students admitted under the new integrated process appeared more motivated to pursue medicine as evidenced by a much lower year 1 discontinuation rate of <2% for the 2003–2005 cohorts compared to 6-10% for cohorts admitted in the years up to 2002 selected on UAI only.

### Divergent validity

Total and dimension-item subtotal scores were analysed for divergent validity as evidenced through low and/or inverse Pearson correlation coefficients. For interview, UMAT and UAI total scores, Pearson correlation coefficient was -0.17. For UAI, interview dimension-items and UMAT dimension-items, the correlation coefficient was 0.30. The higher correlation coefficients for the UAI, interview dimension-items and UMAT dimension-items stem from higher inter-item correlations between UAI and the UMAT dimension-items ‘Reasoning Skills’ and ‘Non-verbal Reasoning Skills’. These latter inter-correlations while significant (*r* = 0.32, *p* < 0.001; *r* = 0.31, *p* < 0.001), were regarded as unlikely to influence regression analysis.

### Correlation coefficients

Pearson correlation coefficients of admission total scores by performance scores are presented in Table 2. UAI correlated significantly with all types of performance scores [WAM phase 1, 0.53 (*p <* 0.01); WAM phase 2, 0.18 (*p <* 0.01); WAM phase 3, 0.28 (*p <* 0.01); WAM final, 0.45 (*p <* 0.01); knowledge examination phase 1, 0.43 (*p <* 0.01); knowledge examination phase 3, 0.30 (*p <* 0.01)], except for clinical skills exam scores in both phase 1 and phase 3. UMAT total score correlated with WAM phase 1 (0.15, *p <* 0.01) and knowledge examination phase 1 (0.12, *p* < 0.05) scores. The interview significantly correlated with clinical skills for phase 1 only (0.19, *p <* 0.01). Total admission scores correlated with WAM phase 1 (0.23, *p <* 0.01), WAM program final (0.15, *p* < 0.05) and knowledge examination scores phase 1 and 3 (0.13, *p <* 0.01 and 0.18, *p <* 0.01).

**Table 2:** Pearson correlation coefficients for total admission scores and performance scores

		**WAM**	**WAM**	**WAM**	**WAM program final**	**Clinical skills**	**Clinical skills Phase 3**	**Knowledge exam Phase 1**	**Knowledge exam**
		**Phase 1**	**Phase 2**	**Phase 3**					
						**Phase 1**			**Phase 3**
		**N = 277**	**N = 283**	**N = 274**	**N = 274**	**N = 301**	**N = 279**	**N = 301**	**N = 279**
UAI	Pearson’s r	0.528	0.179	0.283	0.448	0.112	0.022	0.427	0.298
	Significance (2-tailed)	**<0.001**	**0.002**	**<0.001**	**<0.001**	0.058	0.719	**<0.001**	**<0.001**
UMAT	Pearson’s r	0.148	0.012	-0.030	0.059	-0.056	-0.121	0.123	0.064
	Significance (2-tailed)	**0.014**	0.834	0.625	0.331	0.348	**0.044**	**0.033**	0.290
Interview	Pearson’s r	-0.058	-0.021	-0.013	-0.023	0.193	0.076	-0.054	0.013
	Significance (2-tailed)	0.332	0.720	0.826	0.709	**0.001**	0.205	0.348	0.825
Total Admission Score^1^	Pearson’s r	0.230	-0.261	-0.101	0.151	0.109	0.013	0.131	0.183
	Significance (2-tailed)	**<0.001**	**<0.001**	0.095	**0.012**	0.065	0.831	**0.022**	**0.002**

^1^Total admission score = combined weighted scores of the three components. Significant P-values are boldfaced.

### Correlation coefficients for old selection process and new selection process with UAI

Students admitted in 2002 based on UAI alone comprised a cohort with a very narrow UAI range of 0.25 (99.75 to 100). Thus, correlations between entry criteria and educational outcomes were not considered valid. The 2003 cohort admitted under the integrated process had a UAI range of 5 (95 to 100). For this cohort, significant correlations (*p* < 0.01) were evident between UAI and year 2 average (r = 0.37), year 4 average (r = 0.52), year 6 average (r = 0.38) and overall program average (r = 0.52).

### Regression analysis

#### WAM

The amount of variance in WAM phase 1 accounted for by predictors (adjusted R^2^) was 41.0% and decreased to 31.8% by the end of the program (see Table 3). Significant predictors for both WAM phase 1 and WAM program final scores were UAI (*p <* 0.001) and the interview dimension ‘cope with uncertainty’ (*p <* 0.01, phase 1; *p <* 0.05, program final). The ‘cope with uncertainty’ interview dimension showed a negative prediction for WAM phase 1 and WAM program final scores. The interview communication skills dimension was a significant predictor of WAM program final only (*p <* 0.01). Gender (*p <* 0.05, female) and COB (*p <* 0.05, European descent) were also significant predictors for WAM phase 1, with only gender (*p <* 0.01, female) maintaining statistical significance for WAM program final. The ‘cope with uncertainty’ dimension decreased its unique contribution to WAM variance (*sr*^2^) from phase 1 to program final [2.4% to 1.9% unique variance] and gender maintained its unique variance [2.8%].

**Table 3:** Regression models of the relationship between admission scores (dimensions), demographic variables and performance scores

	**WAM Phase 1**	**WAM program final**	**Clinical skills Phase 1**	**Clinical skills Phase 3**	**Knowledge exam Phase 1**	**Knowledge exam Phase 3**
	**N = 243**	**N = 240**	**N = 251**	**N = 245**	**N = 271**	**N = 245**
Adjusted R^*2*^ (%)	41.0	31.8	17.2	16.7	22.0	19.3
Gender (male = 0/female = 1)						
Beta	0.136^1^	0.180^1^	0.176^1^	0.198^1^	-0.049	0.105
*р*-value	**0.012**	**0.002**	**0.005**	**0.002**	0.407	0.094
*sr*^*2*^ (%)	2.848	2.789	2.660	3.312	0.203	0.941
COB 2 (Euro-derived = 0/EastAsian = 1)						
Beta	-0.137^2^	-0.116	-0.184^2^	-0.230^2^	-0.071	-0.028
*р*-value	**0.015**	0.057	**0.005**	**0.001**	0.243	0.669
*sr*^*2*^ (%)	1.464	1.040	2.650	4.080	0.397	0.063
COB 3 (Euro-derived = 0/Other = 1)						
Beta	-0.042	-.0.005	-0.068	-0.009	-0.058	0.023
*р*-value	0.423	0.929	0.278	0.884	0.320	0.715
*sr*^*2*^ (%)	0.152	0.003	0.397	0.008	0.292	0.044
Education 1 (Group 1 = 0/Group 2 = 1)						
Beta	0.027	0.068	0.048	-0.024	0.058	0.019
*р*-value	0.654	0.292	0.498	0.740	0.376	0.791
*sr*^*2*^ (%)	0.048	0.314	0.152	0.036	0.230	0.023
Rurality (rural = 0/non-rural = 1)						
Beta	-0.132	-0.111	-0.022	0.115	-0.092	0.046
*р*-value	0.051	0.125	0.787	0.153	0.218	0.563
*sr*^*2*^ (%)	0.941	0.672	0.026	0.706	0.436	0.109
UAI						
Beta	0.599	0.513	0.230	0.212	0.469	0.378
*р*-value	**<0.001**	**<0.001**	**<0.001**	**<0.001**	**<0.001**	**<0.001**
*sr*^*2*^ (%)	31.136	22.753	4.580	3.920	18.923	12.461
UMAT 1: verbal reasoning						
Beta	0.075	0.052	-0.037	0.057	-0.021	0.115
*р*-value	0.175	0.383	0.565	0.388	0.725	0.077
*sr*^*2*^ (%)	0.449	0.221	0.109	0.260	0.036	1.04
UMAT 2: understanding others						
Beta	0.095	0.044	0.109	-0.003	0.050	0.016
*р*-value	0.067	0.435	0.075	0.967	0.380	0.796
*sr*^*2*^ (%)	0.828	0.176	1.061	0.000	0.221	0.023
UMAT 3: nonverbal reasoning						
Beta	-0.044	-0.094	-0.028	-0.079	0.018	-0.064
*р*-value	0.471	0.156	0.690	0.273	0.787	0.366
*sr*^*2*^ (%)	0.130	0.578	0.053	0.410	0.023	0.270
Interview 1: motivation						
Beta	0.053	0.037	0.012	-0.052	0.033	0.012
*р*-value	0.494	0.659	0.888	0.570	0.682	0.890
*sr*^*2*^ (%)	0.116	0.058	0.006	0.109	0.048	0.006
Interview 2 – communication						
Beta	0.132	0.206	0.264	0.234	0.120	0.273
*р*-value	0.064	**0.007**	**0.002**	**0.006**	0.119	**0.001**
*sr*^*2*^ (%)	0.846	2.074	3.386	3.312	0.714	3.686
Interview 3: empathy						
Beta	-0.013	-0.049	-0.113	-0.121	-0.040	0.035
*р*-value	0.857	0.524	0.174	0.150	0.609	0.675
*sr*^*2*^ (%)	0.008	0.116	0.624	0.706	0.078	0.058
Interview 4: self-awareness						
Beta	0.103	0.058	0.040	-0.069	0.072	-0.020
*р*-value	0.187	0.497	0.664	0.458	0.397	0.827
*sr*^*2*^ (%)	0.423	0.130	0.063	0.185	0.212	0.017
Interview 5: responding to diversity						
Beta	0.044	0.047	0.130	0.098	0.010	-0.065
*р*-value	0.481	0.482	0.072	0.182	0.878	0.365
*sr*^*2*^ (%)	0.123	0.144	1.082	0.608	0.006	0.270
Interview 6: cope with uncertainty						
Beta	-0.253	-0.223	-0.063	-0.051	-0.158	-0.177
*р*-value	**0.002**	**0.011**	0.498	0.594	0**.**060	0.059
*sr*^*2*^ (%)	2.434	1.850	0.152	0.096	1.04	1.188

^1^Students categorised as female result in criterion increase.

^2^Students COB categorised as European/European-derived result in criterion increase. Significant P-values are boldfaced.

#### Clinical skills

The amount of variance in clinical skills phase 1 accounted for by predictors (adjusted R^2^) was 17.2% and decreased by phase 3 to 16.7% (see Table 3). Significant predictors for both clinical skills phase 1 and 3 scores were UAI (*p <* 0.01) and the communication skills dimension (*p <* 0.01). Gender (female, *p <* 0.01) and COB (*p <* 0.01, European descent) also significantly predicted clinical skills for phase 1 and 3. UAI scores and the communication skills dimension accounted for the highest unique variances (*sr*^2^) towards clinical skills phase 1 at 4.6% and 3.4%, decreasing to 3.9% and 3.3% by phase 3. Gender (female) and COB (European descent) both accounted for 2.7%% variance in phase 1 increasing to 3.3% and 4.1%, respectively.

#### Knowledge examinations

The amount of variance in knowledge examination scores phase 1 accounted for by predictors (adjusted R^2^) was 22.0% and decreased to 19.3% by phase 3 (see Table 3). UAI was a significant predictor for both phase 1 and 3 (*p <* 0.01). The communication skills dimension (*p <* 0.01) significantly predicted phase 3 scores. UAI accounted for the highest unique variance (sr^2^) (18.9% for phase 1, decreasing to 12.5% by phase 3). The communication skills dimension accounted for 3.7% unique variance of phase 3.

#### Effect of rural-entry students

When regression analyses were performed excluding students admitted under the rural entry scheme, there were no significant changes in any statistical outcomes reported compared to analyses of all local-entry students (data not shown).

## Discussion

This study aimed to evaluate an integrated selection process for the Medicine Program introduced at UNSW by determining the predictive validity of the three selection methods; UAI, UMAT and structured interview, controlling for student gender, COB, educational background and rurality. Divergent validity was also examined and results indicate acceptable levels of divergence between UAI, UMAT and the interview.

Predictive validity findings reported were mixed; UAI was the strongest predictor of WAM and knowledge-based outcomes. Although demonstrating a small predictive value, the communication skills dimension was statistically significant in predicting clinical capability, as well as final WAM outcomes, and combined knowledge outcomes for the final two years of study. An unexpected finding was that the ‘cope with uncertainty’ dimension scored during interview had negative predictive value for WAM outcomes. The UMAT was not a significant predictor of any outcome. Female gender and European or European-derived COB presented somewhat consistently as statistically significant predictors of WAM and clinical competency despite having relatively small predictive values.

### Prior academic achievement

Consistent with previous findings, prior academic achievement was the strongest predictor of overall program and knowledge-based outcomes [5,9,10,16,29]. This finding reinforces the use of academic performance as a robust and valuable component of the selection process. Prior academic achievement also demonstrated small yet significant predictive values for clinical skills.

### UMAT

Consistent with previous studies, the UMAT and its dimensions showed poor prediction of outcomes [5,14]. Nevertheless, as discussed below the UMAT may serve an important function to help differentiate amongst a large number of applicants with high academic performance. Evaluating the validity of this process was not possible in this study and requires further research.

### Interview

Of the selection methods, the communication skills dimension emerged as one of the strongest predictors of clinical outcomes, but on the other hand its predictive and unique variance values were relatively small. The communication dimension predicted final WAM outcomes and combined knowledge outcomes for the final two years, albeit also with small predictive values. On the surface these two seemingly contrasting statements point to respective ‘for’ and ‘against’ arguments relating to the use of the interview. Yet given the reported issues that the achievement of high predictive values may be thwarted [19], as well as the socially informed policy factors, it is reasonable to suggest that removing the use of the interview based solely on the predictive validity findings of this study is likely to be a hasty decision. The lower year 1 discontinuation (drop-out) rates for the integrated process that includes the interview suggests its value in differentiating between applicants with variable motivations to pursue a medical career. Additionally, the communication dimension predicted final WAM outcomes and combined knowledge outcomes for the final two years, albeit with small predictive values as well.

The assessment of whether a student has developed clinical capability, while fundamental to the Medicine Program, represents a complex and fluctuating endeavour when compared to developing knowledge-based skills. This is because clinical capability is likely to be a multidimensional construct compared to specific scientific knowledge [30]. Such construct complexity arguably has an unstable effect on the validity of ‘clinical skills’ as a criterion, potentially compromising its relationship to predictors. Furthermore, the relationship between the original differentiating function of the interview and UMAT and pursuing high degrees of predictive validity deserves consideration. That is, given the UMAT and interview were originally designed to assist in distinguishing students with very similar high UAI scores, and not for predicting specific or overall program outcomes, placing sole onus on such selection methods to deliver high predictive values alone may be tenuous.

### Demographics

Literature examining demographic effects on outcomes has been largely confined to measures of ethnicity [23], with some recent studies reporting on gender effects [5,11]. This study demonstrated small yet statistically significant effects for gender, and country of birth. Similar to other studies, females outperformed their male counterparts in overall program and clinical skills outcomes [5,31]. Further, consistent with comparable research [22,23], students who were born in European/European-derived countries outperformed students born in East Asia in clinical skills outcomes and overall program outcomes for the first two years of study. Ethnic differences in medical school performance have been found to be a consistent feature in the UK and such findings should not be considered atypical or as a local problem [23]. It is likely these differences relate to wider structural and social class inequalities that to various degrees permeate local level institutions and practices [32,33].

Although measures of educational background and rurality are commonly unexamined within selection method evaluation studies, this study found no effect of these demographics. One interpretation of this finding is that the university environment provides students with uniform learning opportunities that rescind any disadvantage that may come from living in a remote location, as well as educational advantage that may come from secondary school attended. However, there is no evidence that the authors are aware of to support this interpretation. Rather, studies looking at the relationships between secondary school and university performance have suggested to the contrary [34,35]. However, these studies have only examined first year university performance. Another interpretation could be that our methods of school categorisation may have blunted any observable effects. The lack of effect of rural status on outcomes is encouraging and requires replication in future studies to assure this lack of effect.

Findings comparing the 2002 and 2003 cohort data provide tentative support for the use of the new selection methods. Correlation coefficients between UAI and performance scores among the 2003 cohort who were enrolled in the old Medicine Program via the new selection methods were statistically significant for all outcome measures, whereas the very narrow range of UAI in the 2002 cohort selected via UAI scores alone precluded examination of UAI with outcomes. This comparison between students enrolled in the same program yet through different selection methods, combined with lower year 1 discontinuation rates in students enrolled in the new Medicine Program compared to the previous Program, on face value suggests the differentiating function of UMAT and the interview is effective by improving access to a larger range of academically high performing applicants. However, due to the similar conceptual elements present within both predictive validity and correlation analysis, caution is also warranted with this interpretation. Further research would be valuable in exploring in depth the relationship between the original use of selection tools and prediction. Future research would also benefit from undertaking construct matching to assess the construct validity of selection tools [13]. Limitations of the study including, unaccounted confounders, range restrictions in the 2002 cohort, and the relatively small sample sizes within each cohort, should also be considered when interpreting predictive validity findings [20,36].

## Conclusion

This study evaluated the new selection process introduced at UNSW and confirms the importance of prior academic achievement in predicting student performance. Small yet statistically significant effects relating to gender and COB were also found. This demonstrates that demographic variables, including those typically unexamined in medical education research, should be considered and further researched.

Predictive validity findings for the communication dimension of the interview also demonstrated statistically significant effects. Although on the surface, the smaller effect of interview compared to academic performance may lend support for abandoning the use of the interview, we must also consider other factors located both internally and externally to evaluation discourses premised on predictive validity. When these factors are considered, findings suggest the assessment of communication skills through the interview combined with prior academic achievement are valuable components within an integrated student selection process.

## Competing interests

The authors declare that they have no competing interests.

## Authors’ contributions

PLS drafted the manuscript, participated in the design of the study, and performed the statistical analysis. HAS participated in the design, coordination and helped to draft the manuscript. PDJ conceived of the study and participated in its design. AMDC participated in its design and helped to draft the manuscript. AJO helped to draft the manuscript. PGH helped to draft the manuscript. GMV helped to draft the manuscript. HPM conceived of the study, participated in its design and edited the manuscript. All authors read and approved the final manuscript.

## Authors’ information

Paul L. Simpson, BSc (Psych), PhD is a Research Officer, Faculty of Medicine, University of New South Wales, Sydney, Australia.

Helen A. Scicluna MEd, PhD (Medicine) is a Senior Lecturer, Faculty of Medicine, University of New South Wales, Sydney, Australia.

Philip D. Jones MBBS, MHEd, PhD is the Associate Dean (Education) Faculty of Medicine, University of New South Wales, Sydney, Australia.

Andrew M.D. Cole BSc (Hons), MBBS (Hons) is a Conjoint Associate Professor, School of Public Health and Community Medicine, Faculty of Medicine, University of New South Wales, Sydney, Australia.

Anthony J. O’Sullivan MBBS, MD, MHPEd is an Associate Professor in Medicine, Faculty of Medicine, University of New South Wales, Sydney, Australia.

Peter G. Harris MBBS is a Senior Lecturer, Faculty of Medicine, University of New South Wales, Sydney, Australia.

Gary M. Velan MBBS, Grad Dip HEd, PhD is an Associate Professor in Pathology, School of Medical Sciences, Faculty of Medicine, University of New South Wales, Sydney, Australia.

H. Patrick McNeil MBBS, PhD, Grad Dip HEd is a Professor of Rheumatology, South Western Sydney Clinical School, Faculty of Medicine, University of New South Wales, Sydney, Australia.

## Pre-publication history

The pre-publication history for this paper can be accessed here:

http://www.biomedcentral.com/1472-6920/14/86/prepub
